# Strategies for Implementing Palliative Care Services for Cancer Patients in Low- and Middle-Income Countries: A Systematic Review

**DOI:** 10.1177/00469580251325429

**Published:** 2025-04-02

**Authors:** Neema Florence Vincent Mosha, Patrick Ngulube

**Affiliations:** 1 University of South Africa (UNISA), Pretoria, South Africa

**Keywords:** palliative care, cancer patients, patient navigator-led services, massage therapy, caregivers, low and middle-income countries

## Abstract

Palliative care (PC) services are essential for cancer patients, particularly in low- and middle-income countries (LMICs), where cancer-related deaths are disproportionately high. Despite their significance, access to effective PC remains limited in many LMIC settings. This systematic review aims to identify strategies for implementing PC services for cancer patients in these regions, focusing on the challenges faced. A comprehensive search was conducted for peer-reviewed articles published between January 2004 and July 2024, utilizing the databases Web of Science, Scopus, PubMed, and Google Scholar. The Critical Appraisal Skills Program (CASP) assessment tool was employed to evaluate the quality of the studies following the Preferred Reporting Items for Systematic Reviews and Meta-Analyses (PRISMA) guidelines for transparency. Out of approximately 966 818 articles retrieved, only 17 studies met the defined inclusion criteria. The findings highlighted effective strategies for delivering PC services in LMICs, including patient navigator-led programs, telemedicine, and home health care services. The review highlighted several interventions for PC services, including massage, Cancer and Living Meaningfully (CALM), and light therapies. However, it also identified significant challenges, such as the educational levels of caregivers, patient acceptance of PC services, logistical issues, medication side effects, and a preference for traditional healing practices. This systematic review highlights the critical need for effective PC services for cancer patients in LMICs, where cancer-related mortality rates remain alarmingly high. By synthesizing data from various studies, this analysis offers a comprehensive framework for developing successful palliative care initiatives in these regions. It emphasizes the importance of training caregivers of cancer patients to enhance their confidence in delivering palliative care services and counseling patients about the benefits of these services. Utilizing this information can help practitioners and policymakers improve palliative care services, ultimately enhancing the quality of life for cancer patients in LMICs.

## Introduction

Palliative Care (PC) is a specialized medical approach aimed at alleviating the symptoms and stress associated with serious illnesses, thereby enhancing the quality of life (QoL) for patients and their families.^1^ PC encompasses a range of services, including addressing physical symptoms such as pain, nausea, and fatigue, implementing strategies to improve comfort, assisting patients and families in understanding treatment options, facilitating discussions about goals of care and individual preferences, and coordinating with various healthcare providers to ensure comprehensive care.^2,3^ PC services are offered in diverse settings, such as hospitals, patients’ homes, and dedicated facilities.^4,5^ According to Luyirika et al,^6^ these services are typically delivered by a team consisting of medical practitioners, family members, and volunteers dedicated to supporting individuals facing serious illnesses. Research has shown that early interventions in specialized PC can significantly enhance patient satisfaction, mood, healthcare utilization, and overall survival.^7^ For example, patients receiving early in-home PC alongside standard treatment have reported fewer emergency department visits, reduced hospital admissions, and lower medical costs.^8^ PC caters to a broad spectrum of patients, including cancer patients, focusing on ensuring comfort and improving the end-of-life experience.^9^ Given the well-documented benefits of PC in oncology, particularly in managing symptoms and clarifying the prognosis of terminal illnesses, there is growing advocacy for the early integration of PC for cancer patients.^10^

The World Health Organization (WHO) underscores the importance of incorporating PC into cancer management from the point of diagnosis, ensuring comprehensive support throughout the illness trajectory.^11^ This interdisciplinary approach involves a range of healthcare professionals who address physical symptoms and psychological, social, and spiritual needs.^1,12^ Integrating cancer-related PC is crucial for delivering holistic, high-quality cancer care, particularly in low and middle-income countries (LMICs).^13^ Brant and Silbermann^12^ highlighted that these countries face a disproportionately high burden of cancer deaths, with 70% of global cancer fatalities occurring in LMICs. Annually, approximately 5 million individuals in these regions die from cancer, representing about 10% of the 50 million total deaths in these nations.^13^ Currently, out of 7 million cancer deaths worldwide, 5 million occur in LMICs.^13^ Initiatives such as the Breast Health Global Initiative (BHGI)^14^ and the National Comprehensive Cancer Network (NCCN) guidelines^15^ support the early integration of PC services into oncology, aiming to improve QoL for patients with advanced breast cancer in LMICs. Several factors hinder progress in healthcare, particularly in LMICs. There is a notable shortage of trained healthcare providers and limited access to pain medications, which can affect attitudes toward end-of-life care.^14,15^ Barriers such as insufficient access to early detection and effective cancer treatments also contribute to existing disparities.^15,16^ Although PC services expand globally, their integration and quality in LMICs often fall short.^12^ A key issue is ensuring that PC teams can effectively reach all population.^16^ While ongoing research and initiatives in primary care are taking place in these regions, data gaps continue to pose challenges.^12^ This review aims to compile evidence on effective strategies to enhance PC services in LMICs, identify necessary interventions for delivering these services effectively, and address the challenges that impede access to PC for cancer patients aged 18 to 88 years.

## Methodology

The methodology for this review aims to evaluate research on strategies for implementing PC services for cancer patients in LMICs. It follows the PRISMA guidelines,^17^ as detailed in the checklist in Table 3 of the Supplemental Appendices, which ensures the quality and transparency of systematic reviews. The following steps were taken based on this checklist.

### Research Question

What effective strategies and necessary interventions can be employed to implement PC services for cancer patients in LMICs, and what challenges hinder the delivery of these services in these regions?

### Search Methods

The search strategy was designed to retrieve published research articles published between 2004 and 2024. The search used CINAHL, PubMed, Medline, Scopus, Web of Science (WoS), and Google Scholar databases. Boolean operators “AND” and “OR” were used to narrow the search terms.

### Search Terms

We used the following search terms: Cancer AND Cancer patient OR “Cancer patient* data” AND “PC” OR “PC service*” OR “PC” OR “Palliative therapy” OR “Palliative treatment” OR “Palliative medicine” OR “End of life care” OR “Comfort care” AND Effectiveness OR “Efficacy” OR “Influence” AND Low- and middle-income countries OR “LMICs” OR “Developing countries.”

### Inclusion and Exclusion Criteria

The inclusion criteria for this systematic review focused on studies involving cancer patients receiving PC services in LMICs, encompassing various interventions such as therapeutic approaches (eg, massage, CALM, light therapies) and supportive care strategies (eg, telemedicine, home care). Only peer-reviewed articles published between January 2004 and July 2024 that reported on the effectiveness of PC services in terms of patient QoL, symptom management, caregiver support, and overall satisfaction were included. Conversely, studies not focusing on cancer patients, those conducted in high-income countries, or solely addressing curative treatments were excluded, along with non-peer-reviewed articles, opinion pieces, and publications not in English. This approach ensures that the review encompasses relevant, high-quality research that contributes meaningful insights into PC implementation for cancer patients in LMICs.

### Data Extraction and Management

After identifying and reviewing the final studies, a data extraction framework was created and refined based on the Template for Intervention Description and Replication (TIDieR).^18^ The data extraction concentrated on essential information, including author(s) and publication date, study setting, study design, data collection and sampling, main results, PC setting, and overall impact. The primary author (NFVM) extracted general review information, while the second author (PN) independently extracted the outcome data. Table 1 in Supplemental Appendix 1 illustrates data extraction and management.

### Quality Assessment

We used CASP to assess the quality of the reviewed studies, whereas out of 17 identified studies, RCTs (4), qualitative (6), and cross-sectional (7). Table 2 in Supplemental Appendix 2 provides the assessment of methodological quality for prevalence as per the Critical Appraisal Skills Programme (CASP) checklist.

## Results

### Description of Studies

**Figure 1.**
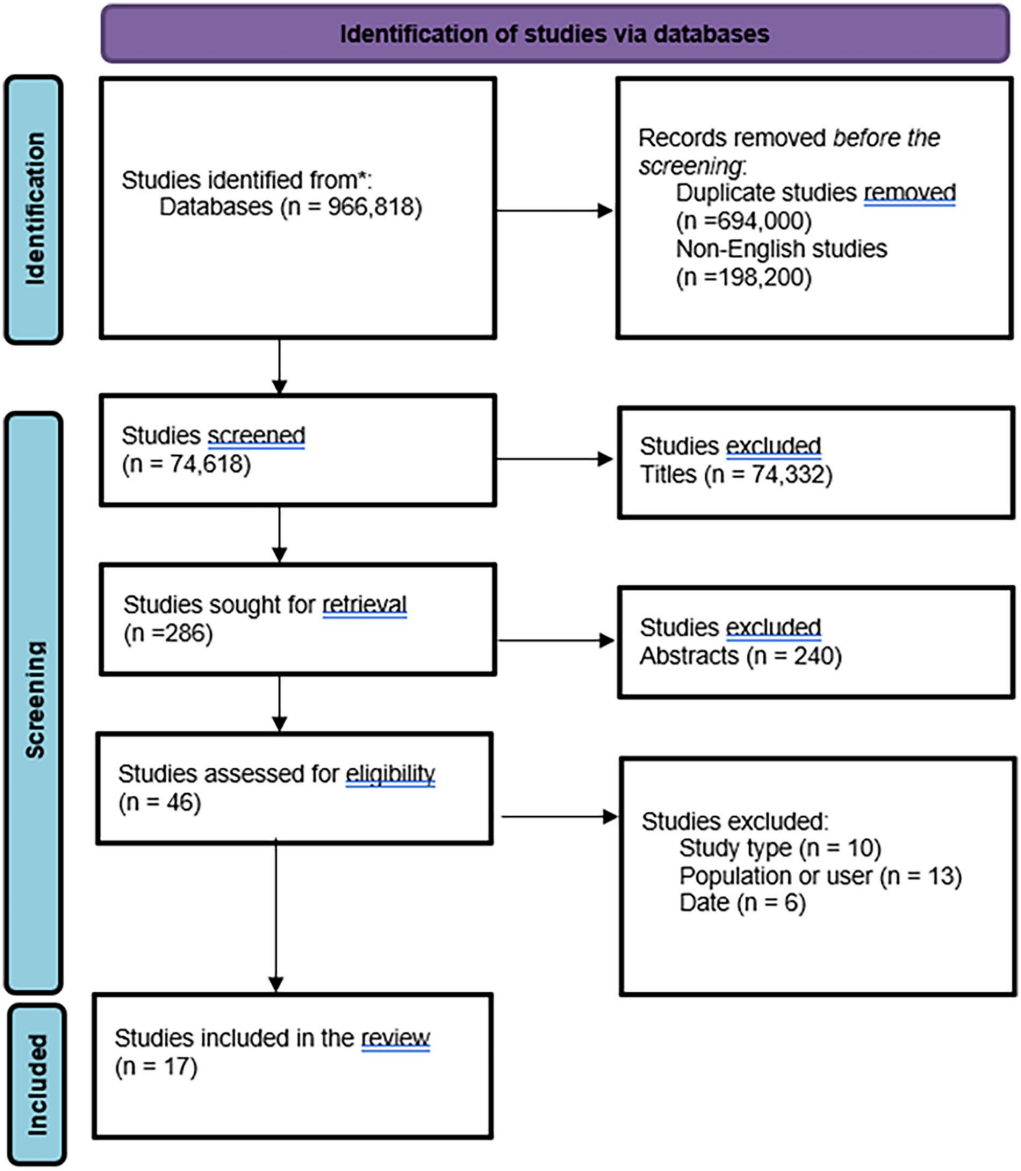
PRISMA flow diagram-search process.

The searches were undertaken between July to September 2024. Searches returned a total of 966 818 studies. All studies retrieved were imported into CADIMA, an online tool supporting the conduct and reporting of systematic reviews and systematic maps for screening purposes, which reached 17 studies for analysis. The complete search process is detailed in the PRISMA diagram (Figure 1).

### Effective Strategies to Implement PC Services

PN-led initiatives, spiritual well-being (SWB) programs, telemedicine services, medical and social care support, home care services, community PC services, and home-based care^19-21,22-26^ were identified in this review. A PN-led initiative is an effective PC service delivered by healthcare professionals to overcome barriers to accessing PC services.^19^ This initiative increased the completion of advance directives |(AD) and reduced the number of cancer patients in Mexico.^19^ In Bangladesh, Chowdhury et al^20^ introduced a community home-based PC model that involved training patients’ relatives and local volunteers. In contrast, some nurses voluntarily provided home-based care for patients who were unable to attend hospitals.^20^ This reflects a commitment to personalized and compassionate care during the end-of-life stage.^24^ Mughrabi et al^25^ highlighted the role of a multidisciplinary team in delivering home care services at the King Hussein Cancer Center involving physicians, nurses, and clinical pharmacists facilitated PC services.^25^ Biswas et al^26^ found a strong negative correlation between perceived social support and psychological issues such as depression and anxiety among cancer patients. Guo et al^21^ emphasized the transformative potential of telemedicine for family caregivers in end-of-life care.

### Interventions in PC Services

The current review identified several interventions, including massage, Cancer and Living Meaningfully (CALM), Wait-list Control (WLC) therapies, as well as Bright White Light (BWL) and Dim Red Light (DRL).^27-29^ Also, AHSCP medical and social care, home-based care, and community-based support are crucial for enhancing the well-being of cancer patients.^20,23-26^ Miladinia et al^27^ studied the effectiveness of massage therapy within PC programs, focusing on a specific cluster of oncology-related symptoms. Zhang et al^28^ investigated the feasibility of CALM and WLC therapies for patients with metastatic breast cancer. Afessa et al^30^ noted that cancer patients with higher education levels could better understand healthcare instructions, facilitating their navigation of the healthcare systems. A sense of meaning was identified as a valuable resource for advanced cancer patients coping with insomnia and fatigue. Zhang et al^28^ reported that both CALM and WLC therapies enhanced patients’ sense of life completion at the end of life.^28^ The BWL program significantly reduced fatigue levels by 25.09%, while the DRL used in the control group yielded a smaller reduction of 4.08%.^29^ The efficacy of BWL applied at a luminescence of 10 000 lux was underscored by its impact on both fatigue and sleep quality.^29^ Yang et al^22^ found distinct dimensions of SWB related to the patient’s experiences of depression, anxiety, and pain. Gontijo Garcia et al^31^ observed a significant negative relationship between family support and levels of anxiety, stress, and depression. Social and spiritual support were shown to provide relief to cancer patients beyond what medications alone can offer, with social backing being particularly impactful in regions like Bangladesh.^27^

Appiah et al^24^ emphasized that nurses often discuss the pain medications they prescribe, focusing on their effectiveness in alleviating pain, minimizing addiction risks, and considering potential side effects. Afessa et al^30^ found that patients experiencing side effects of these medications were 3.5 times more likely to utilize PC services. Chowdhury et al^20^ suggested that increasing community engagement and understanding of PC could improve outcomes for cancer patients, including their QoL and symptom burden. Afessa et al^30^ noted that patients living near PC centers and with higher education levels were more likely to attend appointments as they better understood the side effects that PC services address. Fetene et al^32^ recommended that Hawassa University Comprehensive and Specialized Hospital develop strategies to enhance PC service utilization.

### Challenges Associated With the Provision of PC Services in LMICs

Soto-Perez-de-Celis et al,^19^ found limited financial resources negatively impacted patients’ willingness and ability to engage with interventions in Mexico. Many patients often opt for curative treatments from conventional medicine or traditional healers.^33^ Mental health challenges hinder the utilization of PC services.^22,26,28,31^ Nausea and fatigue worsen mental health challenges, limiting patient engagement.^34^ Younger cancer patients experience depression and anxiety, which hinder seeking PC services.^22,26,28,31^ Insufficient knowledge about PC approaches can lead to misunderstandings and inadequate patient support.^29^ Infrastructure-related issues, including a shortage of human resources, significantly impede the provision of home-based PC services.^26,27,30-33^ Geographical disparities play a crucial role in accessing PC services.^23^ Misconceptions and inadequate education about PC create substantial barriers to effective service delivery.^24,27,33^ Communication gaps between PC providers, patients, and their families exacerbate delays in service provision.^33^ Misunderstandings about the scope of PC services, often mistakenly viewed as solely related to pain management or end-of-life care, can foster anxiety and confusion among patients and their families.^23^ The absence of community volunteers and health extension workers limits patient connections to necessary services^33^ Negative public perceptions of PC and a shortage of resources, such as treatment rooms and trained staff, were also noted.^24^ Nurses, in particular, express frustration over long wait times for treatments and the inability to address resource deficiencies, especially in rural areas where access is already compromised.^24^ Overall, the challenges of providing PC services in LMICs are complex and multifaceted.

## Discussion

The review advocates for LMICs to implement strategies such as PN-led multidisciplinary teams, also noted by Gaertner et al.^34^ A notable finding was the AD completion rate of 44%, which is higher than in control groups.^35^ The efficacy of massage therapy demonstrated effectiveness in alleviating cancer-related symptoms like pain, fatigue, and sleep disturbances.^27^ Cronfalk et al^36^ found soft tissue massage provided a sense of existential respite, allowing temporary relief among patients. The review emphasized the integration of BWL and DRL therapies into nursing care to enhance fatigue and sleep disorder management.^29^ Wu et al^37^ noted trends toward increased sleep duration in BWL patients, which is also linked to a sense of peace with improved physical and psychological outcomes.^38^ The review found many medications prescribed to patients were appropriate.^25^ Hospital nurses adhered to WHO pain management guidelines, highlighting the efficacy of analgesics, particularly morphine, for cancer-related pain.^39-41^ However, the review identified a preference among patients for traditional healers over conventional medicine.^32,33^ This trend is corroborated by a multicounty study indicating that patients often seek traditional healers and herbalists for cancer treatment.^41,42^ High levels of depression, stress, and anxiety were reported among both patients and caregivers in the reviewed articles.^22,26,28,31^ Caregivers often experience greater distress than patients with cancer.^43^ Bužgová et al^44^ found anxiety and depression are prevalent among patients in advanced stages receiving PC, often leading to panic or maladaptive behaviors.^45^ Pain was directly linked to depression, with Azevedo et al^46^ noting a strong correlation between pain levels, depression, and functional capacity. Perceived social support appeared to buffer the effects of pain and depression, with patients reporting lower levels of depression when feeling supported.^26^ Fisher et al^47^ and Galloway et al^48^ also highlighted the relationship between social support, pain, and depressive symptoms. In providing holistic care, nurses prioritized SWB to foster hope and improve QoL^24^ which was also emphasized in several studies.^49,50^ The WHO recommends integrating SWB into PC services to reduce anxiety and enhance comfort.^51^ Workshops on SWB support healthcare providers working with terminally ill patients.^52^

Telemedicine emerged as a valuable tool for connecting caregivers with healthcare professionals, particularly in emergencies, thus overcoming geographic barriers.^21,23^ Telemedicine enhances access to limited resources.^53,54^ Most caregivers preferred web-based resources^55^ despite challenges such as difficulty conveying empathy through technology.^56^ Gaertner et al^34^ highlighted the need to focus on patient-reported symptoms to facilitate real-world implementation of care. Busch et al^57^ outlined key factors influencing the caregiver-patient relationship, including respect for dignity, individuality, humanity, and empathy. Barberia et al^58^ emphasized the potential of virtual reality-based games to simulate end-of-life experiences, offering innovative strategies to help caregivers support patients in facing death more comfortably. The current review found a lack of communication among AHSCPs, patients, and their families.^23^ Lemus-Riscanevo et al^59^ noted that inadequate communication delays critical support for cancer patients. Social networks like Twitter and Facebook support open discussions about PC among patients, families, and healthcare professionals.^60^ The review found geographical barriers contributing to inequities in PC provision, particularly for patients with incurable cancer in rural areas of Colombia.^23^ Pastrana et al^61^ suggested the need for public policies and local guidelines advocating for PC integration through multidisciplinary teams. Calvache et al^62^ noted PC programs in capital cities like Bogotá and regions such as Antioquia, Valle del Cauca, and Atlántico. However, rural areas, such as Cauca, have a similar number of patients requiring PC services.^63^ The disparity in PC availability is linked to the socio-economic development of the regions, with less developed areas offering more limited services.^63^ The review also highlighted that cancer patients with higher education levels are more likely to accept and understand PC services,^30^ a finding that Weiss et al^64^ corroborated.

This systematic review has several limitations that may impact the findings and their generalizability. Firstly, although the search strategy was comprehensive, it did not include all relevant studies, especially those published in languages other than English or found in less accessible databases. Secondly, the review included only 17 studies that met the inclusion criteria, which restricted the scope and diversity of the analyzed data. This small sample size may not adequately represent the variety of experiences and challenges associated with palliative care (PC) in low- and middle-income countries (LMICs).

Thirdly, many studies relied on self-reported data, which could introduce bias and compromise the reliability of the findings since patients and caregivers may have differing perceptions of PC services. Furthermore, the review primarily focused on specific therapies and interventions, potentially overlooking the full range of PC practices available in LMICs. Lastly, while cultural factors influencing the acceptance and utilization of PC services were acknowledged, they were not thoroughly explored. This lack of exploration may limit the understanding of patient preferences and behaviors in different cultural contexts.

## Conclusion

This systematic review highlights the critical need for effective PC services for cancer patients in LMICs, where cancer-related mortality remains alarmingly high. Despite the significant benefits of PC, access to these services is often limited due to various challenges, including educational gaps among caregivers, patient acceptance issues, logistical barriers, and a reliance on traditional healing practices. The review identified several effective strategies for implementing PC services, such as patient navigator-led initiatives, telemedicine, and home care approaches, alongside therapeutic interventions like massage and CALM. However, to maximize the impact of these strategies, it is essential to focus on training caregivers and educating patients about the benefits of PC services. Moving forward, ongoing research is vital to evaluate the effectiveness of different implementation strategies and to develop best practices tailored to the unique contexts of LMICs. By addressing these challenges and promoting collaboration among healthcare stakeholders, the delivery of PC services will be enhanced, ultimately improving the QoL for cancer patients and their families in these underserved regions.

## Supplemental Material

sj-docx-1-inq-10.1177_00469580251325429 – Supplemental material for Strategies for Implementing Palliative Care Services for Cancer Patients in Low- and Middle-Income Countries: A Systematic Review Supplemental material, sj-docx-1-inq-10.1177_00469580251325429 for Strategies for Implementing Palliative Care Services for Cancer Patients in Low- and Middle-Income Countries: A Systematic Review by Neema Florence Vincent Mosha and Patrick Ngulube in INQUIRY: The Journal of Health Care Organization, Provision, and Financing
