# Supplementary material for: Strategies for Implementing Palliative Care Services for Cancer Patients in Low- and Middle-Income Countries: A Systematic Review
Source: Inquiry. 2025 Apr 2;62:00469580251325429. doi: 10.1177/00469580251325429 (PMC11967213; doi:10.1177/00469580251325429)
Supplement: sj-docx-1-inq-10.1177_00469580251325429 – Supplemental material for Strategies for Implementing Palliative Care Services for Cancer Patients in Low- and Middle-Income Countries: A Systematic Review [file sj-docx-1-inq-10.1177_00469580251325429.docx]

**APPENDICES**

**Table 1: Data extraction and management**

| SN | Author(s) | Study setting | Study design | Data collection and sampling | Main results | PC setting | Overall impact |
| --- | --- | --- | --- | --- | --- | --- | --- |
| 1 | Soto‐Perez‐de‐Celis et al. [18] | Mexico | RCTs were carried out from August 2017 to April 2018 at a public hospital in Mexico City. | After randomization, 134 cancer patients in Mexico completed validated questionnaires using electronic tablets. | Of 134 randomized patients, 67 received PN-led initiative and 67 usual cares. Supportive care was given to 74% in PN versus 24% in usual care. After 12 weeks, 10% of PN patients had moderate to severe pain versus 33% in usual care. | A PN-led multidisciplinary intervention significantly enhanced access to supportive and PC for Mexican patients with metastatic solid tumours. | PN-led enhances access to early supportive care, advance care planning, and pain management for patients with advanced cancer in LMIC settings. |
| 2 | Miladinia et al. [19] | Iran | This 7-arm RCT involved weekly massages over 4 weeks (August 13, 2021, to January 12, 2022), with durations of 15, 30, or 60 minutes and frequencies of 2 or 3 times per week, followed by a 4-week follow-up. | A sample size of 217 subjects was determined for comparing study groups, using a 5% significance level, 80% power, an effect size of 0.70, seven groups (7-arm), and eight repeated measures over time. | The findings revealed that dose escalation enhances the efficacy of massage therapy for the pain-fatigue-sleep symptom cluster. While 60-minute sessions were more effective, 30-minute sessions may be more practical due to their lower cost and reduced time commitment. | The study tested six massage doses, varying by frequency (2 or 3 sessions weekly) and duration (15, 30, or 60 minutes), using Slow-Stroke Back Massage (SSBM) with gentle, rhythmic palm movements. | The effectiveness of massage therapy in PC remains uncertain. This study integrated massage therapy into palliative cancer care programs and represents the first dose-response trial examining the use of massage therapy in this context. |
| 3 | Chowdhury et al. [20] | Bangladesh | A longitudinal observational study was conducted from January 2020 to December 2021. | Thirty-nine cancer patients received comprehensive PC in the NPC group, while thirty-one patients received standard care in the DO group. The NPC group had more older patients and women with higher education. At follow-up, the NPC group showed significant improvements in overall QoL and various domains. | Comprehensive PC significantly improved QoL (p<0.05) in 10-14 weeks. The oncology control group showed fewer improvements, with increased disability and pain. PC patients experienced less decline in palliative performance and neuropathic pain compared to the control group. | The "Compassionate Narayanganj" program created and launched an innovative community home-based PC service, centered at the NCC, along with an emergency telephone hotline to meet the community's unmet PC needs. | Palliative treatment significantly improved pain, drowsiness, shortness of breath, and overall well-being. The most commonly used medications were anti-emetics and proton pump inhibitors (PPIs). This research shows that community PC can enhance cancer patients' QoL, highlighting the need for such a model in Bangladesh. |
| 4 | Zhang et al. [21] | China | The study conducted a RCT to assess the feasibility and preliminary efficacy of CALM and WLC therapies. All patients were recruited from Peking University Cancer Hospital, including outpatients from the psycho-oncology department and inpatients from the breast oncology department, between January 2022 and March 2023. | Thirty-six patients were randomly assigned to two groups, with 34 completing all assessments. The Nine-Item Patient Health Questionnaire (PHQ-9) assesses depressive symptoms, with a score of ≥10 indicating moderate depression. | Patients meeting the inclusion criteria were randomly assigned to the CALM group or the Wait-list Control. The mean age was 47.26 years, with most being unmarried, city dwellers, and medically insured. | CALM and WLC therapies were administered. CALM therapy significantly alleviated psychological burden and improved the QoL in patients with metastatic breast cancer. | The study offers preliminary evidence supporting the efficacy of CALM therapy in alleviating depression, distress, death anxiety, and suicidal ideation in Chinese patients with metastatic breast cancer. |
| 5 | Guo et al. [22] | China | A descriptive phenomenological approach was conducted. One-on-one semi-structured interviews were conducted with participants from November to December 2022. Colaizzi’s method was used to analyse the interviews. | Purposive sampling was used to select family caregivers of patients with end-of-life cancer attending a tertiary cancer hospital in China. Semi-structured, face-to-face interviews were conducted by JG in a private room at the hospital, scheduled at the participants' convenience. The interviews lasted between 21 and 36 minutes and were audio recorded with the participants' consent. | Fourteen participants were interviewed, revealing three main themes with ten subthemes: motivation for telemedicine services, including relief from home care and access to professional healthcare; supportive care needs like symptom management and emotional adjustment; and functional expectations of platforms, emphasizing ease of use, online guidance, and personalized reminders. | Telemedicine is rapidly transforming healthcare delivery, particularly in end-of-life care. This study explored the perspectives and preferences of family caregivers for end-of-life cancer patients regarding telemedicine services. It provides a framework for developing a tailored telemedicine-based end-of-life care program that addresses the specific needs of family caregivers in China. | Family caregivers showed interest in telemedicine-based services and identified several care needs prior to utilizing these services. The findings of this study can assist policymakers and healthcare providers in creating more effective and culturally appropriate telemedicine programs that better support family caregivers of patients with end-of-life cancer. |
| 6 | Costa et al.[23] | Brazil | Qualitative analysis was conducted as part of a larger prospective cohort study that enrolled patients with advanced cancer and pain who were hospitalized in the specialized PC Unit of the National Cancer Institute (INCA) from June 2021 to February 2022. | A total of 104 patients were selected for the study, with data collected through face-to-face interviews. Using the validated Brief Pain Inventory, the research examined the correlation between pain and quality of life in advanced cancer patients on their first day in a PC unit. Fatigue, nausea, and physical health showed the strongest correlations with cancer pain. | The participants had a mean age of 53.6 years (SD ± 14.1), predominantly female (65.38%) and white (43.27%). Most completed primary education (67.31%) and reported family incomes up to one minimum wage (55.77%). Significant correlations were found between cancer pain severity and quality of life across all domains, particularly physical health. | Not indicated | The findings show a strong correlation between cancer pain and reduced quality of life in advanced cancer patients receiving PC. Effective symptom management is crucial for optimizing quality of life, enhancing multidisciplinary care, and improving palliative services for this population and their families. |
| 7 | Afessa et al. [24] | Ethiopia | A cross-sectional study design was conducted in this study. | A structured questionnaire was given to 404 participants at Tikur Anbesa Specialized Hospital and Saint Paul’s Hospital from July 4 to August 2, 2022, using ODK-Collect software and systematic random sampling from cancer patient records. | The utilization rate of PC services was 35.4% [95% CI: 31.4, 40.3%]. Patients with a college education were 2.3 times more likely to use PC services. Those living within 23 km were 1.8 times more likely, and those without treatment side effects were 3.5 times more likely to utilize these services. Satisfaction with healthcare increased the likelihood of using PC services by 2.1 times. | Not indicated | The utilization of PC services was notably low based on various factors identified by this study. The study recommended the improvement of PC services for cancer patients, interventions focused on enhancing health education and counselling, early detection and management of treatment of side effects, and the accessibility of PC services. |
| 8 | Gontijo Garcia et al. [25] | Brazil | A cross-sectional study was conducted involving patients with advanced cancer who were receiving PC services. Eligible participants included cancer patients aged 18 years or older with preserved cognitive function who completed the questionnaires | The study used the Hospital Anxiety and Depression Scale (HADS) and the EORTC-C30 quality of life questionnaire. Data were analyzed using R software, with 70 out of 78 invited patients participating, reflecting a 10.2% refusal rate. All instruments were validated for the Brazilian population. | The study found depression in 44.3%, anxiety in 28.5%, and both symptoms in 52.9% of advanced cancer patients receiving PC. Better physical and overall functionality reduced the likelihood of these symptoms, while nausea, fatigue, and appetite loss increased it. | Functioning (including physical and social function) and symptom management are essential for ensuring the best QoL for patients with advanced cancer receiving PC. This underscores the need for effective control and relief of symptoms. | This study revealed a high prevalence of anxiety and depression among patients. Better QoL and functionality were negatively associated with these conditions. Evaluating patients' functioning can help clinicians alleviate symptoms of anxiety and depression, thereby enhancing the dignity of cancer patients in PC. |
| 9 | Yang et al. [26] | China | A cross-sectional study was conducted involving consecutive inpatients admitted to Shengjing Hospital of China Medical University between July 2019 and October 2020. | Patients completed questionnaires on SWB and cancer-related symptoms including insomnia, fatigue, pain, depression, and anxiety upon admission. Linear regression analysis was used to assess the relationship between SWB (meaning, peace, and faith) and symptom distress. | SWB explained 17.8% to 44.4% of the variance in cancer-related symptoms. Meaning negatively correlated with insomnia (β = -0.516) and fatigue (β = -0.563). Peace and faith reduced psychological symptoms, while meaning positively impacted anxiety (β = 0.275). Higher peace levels correlated with lower cancer pain (β = -0.422). | The present study underscores the significance of spiritual care within integrated PC services by identifying the different dimensions of SWB among patients with advanced cancer. | The present study suggests that the distinct dimensions of SWB enable individuals to overcome cancer-related symptoms in a PC unit. Specifically, attaining peace and faith consistently served as positive resources for patients with advanced cancer. |
| 10 | Çelik and Usta Yeşilbalkan [27] | Turkey | This study was a RCT that investigated the effects of BWL at a luminosity of 10,000 lux on fatigue levels and sleep quality in patients, comparing it DRL at a luminosity of less than 50 lux. The study was conducted in the PCU of a university hospital. | The study involved 52 cancer patients, divided into two equal groups in a university hospital's PCU. Participants were randomly assigned based on sex, ensuring balanced allocation. Randomization was managed by a biostatistician using R software, keeping both researchers and patients unaware of the group assignments until light application. | Both groups were monitored for sleep environments and side effects during light application. All patients avoided stimulants before sleep, maintained dim lighting at 24-25°C, and experienced no side effects. Significant fatigue changes were noted over time (p < 0.001). The study group showed a 26.07% improvement in fatigue, while the control group showed no significant differences. | This study investigated the effects of BWL on fatigue levels and sleep quality in cancer patients receiving PC services. BWL is administered in the early morning for 30 to 90 minutes, using high-intensity fluorescent light (10,000 lux) from a light source. It helps regulate the circadian rhythm by stimulating the suprachiasmatic nucleus in the hypothalamus. | This study supports previous findings that BWL effectively manages fatigue and improves sleep quality in cancer patients receiving PC. We recommend assessing these symptoms and promoting BWL use to enhance comfort and QoL for patients. Education for patients, families, and healthcare professionals is essential. |
| 11 | Mendieta et al. [28] | Colombia | Two focus groups were held as part of the World Café method. This approach enhances collective intelligence by fostering open discussions among diverse expert stakeholders about their ideas and experiences. It involves small group discussions centered around predetermined questions aligned with the goals of the World Café. | AHSCPs with experience in PC services were invited through social networks (WhatsApp, Facebook, or email) in two cities in Colombia Bogotá and Popayán in April 2022. Non-probabilistic snowball sampling was used to recruit a diverse group of AHSCPs. | Eighteen AHSCPs participated in the two World Café groups held in Popayán and Bogotá. Through this interactive process, we identified five thematic areas: humanizing care, normalizing PC services at the time of diagnosis, misconceptions related to PC, barriers within the healthcare system, and geographic challenges. | The role of AHSCPs in providing comprehensive, high-quality PC. | This study offers valuable insights into the perspectives of AHSCPs in Colombia regarding barriers to equitable access to PC an area that has been previously overlooked. The study highlighted various barriers and their causes. |
| 12 | Abate et al. [29] | Ethiopia | The study employed an exploratory qualitative design with a deductive approach, whereby patients, caregivers, and professionals were interviewed to gather their perspectives on patient-professional interactions and the organization of the healthcare system. | Interviews were pre-arranged and conducted face-to-face at participant-chosen venues by researchers. Notes and memos were recorded, and the interview guide was refined during data collection from January to September 2020 in Addis Ababa's hospitals. Participants were purposefully selected. | Key barriers to continuous palliative care included opioid scarcity, high turnover, and healthcare worker shortages. Accessibility issues arose from limited diagnostic materials, medication costs, insufficient government support, and home care capacity. Cultural barriers and patient preferences for conventional medicine further impacted care delivery and coordination effectiveness. | Not indicated | In Ethiopia, the continuum of palliative care from health facilities to households is still developing, hindered by issues of availability, accessibility, acceptability, utilization, and effectiveness. Further research is needed to clarify roles, and the health sector must enhance this continuum to meet increasing needs. |
| 13 | Fetene et al. [30] | Ethiopia | A hospital-based cross-sectional study was conducted among women with breast cancer, utilizing a cross-sectional study design. | Data were collected during visits to the oncology unit between August 1 and October 30, 2021. All patients attending that unit were invited to participate in the study. Women aged 18 years or older were included, while critically ill patients were excluded. The study employed a consecutive sampling technique. | This study found that 72 women (59.5%) experienced poorer utilization of PC services, with living in a rural area identified as a contributing factor. The odds of worse utilization were significantly higher in rural areas, with an adjusted odds ratio of 11.82. | The study highlighted the differences in PC service provision between cancer patients living in rural and urban areas. | The study found over half of participants poorly utilized PC services, with rural living as a contributing factor. Despite challenges, Ghanaian oncology nurses effectively deliver essential PC, including pain management and spiritual support. |
| 14 | Appiah et al. [31] | Ghana | A qualitative exploratory-descriptive design was utilized to gain insights into PC nurses' experiences, roles, and the challenges they faced in the PC unit. This approach was selected because it is particularly effective for exploring areas with limited prior research, such as PC services in Ghana. | Researchers created a semi-structured interview guide with open-ended questions to gather detailed information. It included sections on socio-demographics, oncology nurses' roles in palliative care, and challenges faced. Interviews lasted 40 to 60 minutes. | Participants ranged in age from 25 to 40 years, with a higher percentage of females (n = 17, 57%) compared to males (n = 13, 43%). Two main themes emerged from the study: the delivery of PC and the provision of home care services. The current roles of nurses focused on pain management, home care services, spiritual needs, and psychological support. | The study revealed an unexpected finding: some nurses were voluntarily providing home-based care to patients who could no longer receive end-of-life care in the hospital. It was surprising that these nurses felt compelled to volunteer their services for patients who could not afford treatment at home. | The study found over half of participants poorly utilized palliative care services, with rural living as a contributing factor. Despite challenges, Ghanaian oncology nurses effectively deliver essential palliative care, including pain management and spiritual support. |
| 15 | Mughrabi et al. [32] | Jordan | A retrospective observational study was conducted at King Hussein Cancer Center (KHCC), a leading comprehensive cancer center in Jordan. | The study included adult cancer patients who received home care services from January to December 2020. | The study involved 353 patients, median age 64, with 184 (52%) males. About 45.6% had multiple comorbidities, mainly cardiovascular diseases (46.9%). Most patients (94.1%) had solid tumors, with 78.2% metastatic disease. Analysis of 2,707 medications revealed 63.2% were potentially appropriate, including opioids and laxatives. | At KHCC, home care services assist cancer patients with reduced performance status who cannot attend hospital follow-ups. A multidisciplinary team provides consultations for symptom management and referrals to PC services after active treatment ends. | About one-third of medications in home care are potentially avoidable. To optimize prescribing, measures include creating deprescribing guidelines for end-of-life patients, promoting interdisciplinary discussions, and involving patients and families in medication discontinuation decisions. |
| 16 | Biswas et al. [33] | Bangladesh | The study used a cross-sectional survey with 115 advanced cancer patients (stages III and IV) in a Bangladeshi palliative medicine department. Social support was measured by the Multidimensional Scale, and mental health by the DASS-21. | Data were collected via a structured questionnaire from June to November 2023. Relationships between perceived social support, depression, anxiety, and stress were assessed using Spearman correlation and multiple linear regression analysis. | The study included nearly equal male (49.6%) and female (50.4%) participants, averaging 50.7 years. Most reported moderate to high social support, while 78.3% faced anxiety, 77.4% depression, and 70.5% stress. | This study explores perceived social support among Bangladeshi cancer patients and its impact on mental health. Higher social support is associated with lower depression, anxiety, and stress, acting as a buffer against negative emotions, particularly during physical pain. | Perceived social support significantly impacts the mental health of patients with advanced cancer. Integrating psychosocial support early in PC can greatly enhance the mental well-being of these patients. |
| 17 | Kaur et al. [34] | India | The study used descriptive cross-sectional research in Dhanas village, Chandigarh, including all residents. Data collection tools included a screening questionnaire, sociodemographic sheet, clinical profile, Barthel Index, and pain rating scale, analyzed with SPSS version 19. | A total of 10,021 individuals from 884 households in the area were assessed for the need for PC services. A survey was conducted on the entire population of Dhanas village, selected through purposive sampling as it serves as the field practice area for the investigators. Data were collected through a house-to-house survey conducted in March 2019. | The results indicated that the prevalence of the need for PC services was 2 per 1,000 individuals. None of the 19 participants with unmet PC needs were receiving any home or institutional-based services at the time of assessment. Approximately one-fourth of the participants were completely dependent on caregivers for ADL. | The study indicated a high level of demand for PC services among patients in India. | The study concluded that there is a significant need for PC services in the rural community of Chandigarh. This data can be utilized for planning and implementing community-based PC services in the area. |

**Table 2. Assessment of Methodological Quality for Prevalence Studies (CASP Checklist)**

| Checklist for RCT | 1 | 2 | 3 | 4 | 5 | 6 | 7 | 8 | 9 | 10 | 11 | 12 |
| --- | --- | --- | --- | --- | --- | --- | --- | --- | --- | --- | --- | --- |
| Soto‐Perez‐de‐Celis et al. [18] | Y | Y | N | NI | U | NI | Y | Y | Y | NI | NI | - |
| Miladinia et al. [19] | Y | Y | N | NI | Y | Y | Y | Y | NI | N | U | - |
| Zhang et al. [21] | Y | Y | NI | NI | N | U | Y | Y | Y | N | NI | - |
| Çelik and Usta Yeşilbalkan [27] | Y | Y | Y | NI | Y | Y | NI | Y | U | U | NI | - |
| Questions | | | | | | | | | | | | |
| 1. Did the study address a clearly focused research question? | | | | | | | | | | | | |
| 1. Was the assignment of participants to interventions randomised? | | | | | | | | | | | | |
| 1. Were all participants who entered the study accounted for at its conclusion? | | | | | | | | | | | | |
| 1. Were the participants ‘blind’ to intervention they were given? | | | | | | | | | | | | |
| 1. Were the study groups similar at the start of the randomised controlled trial? | | | | | | | | | | | | |
| 1. Apart from the experimental intervention, did each study group receive the same level of care (that is, were they treated equally)? | | | | | | | | | | | | |
| 1. Were the effects of intervention reported comprehensively? | | | | | | | | | | | | |
| 1. Was the precision of the estimate of the intervention or treatment effect reported? | | | | | | | | | | | | |
| 1. Do the benefits of the experimental intervention outweigh the harms and costs? | | | | | | | | | | | | |
| 1. Can the results be applied to your local population/in your context? | | | | | | | | | | | | |
| 1. Would the experimental intervention provide greater value to the people in your care than any of the existing interventions? | | | | | | | | | | | | |
| Checklist for prevalence studies | 1 | 2 | 3 | 4 | 5 | 6 | 7 | 8 | 9 | 10 | 11 | 12 |
| Costa et al. [23] | Y | N | N | NI | Y | Y | Y | Y | NI | N | N | Y |
| Afessa et al. [24] | Y | Y | Y | Y | Y | U | N | Y | Y | U | N | Y |
| Gontijo Garcia et al. [25] | Y | NI | N | N | Y | Y | Y | NI | Y | U | Y | Y |
| Yang et al. [26] | Y | N | Y | NI | Y | Y | N | N | Y | NI | Y | Y |
| Fetene et al. [30] | Y | Y | NI | Y | U | Y | NI | NI | NI | NI | Y | Y |
| Biswas et al. [33] | Y | Y | Y | Y | Y | Y | NI | NI | Y | NI | Y | Y |
| Kaur et al. [34] | Y | Y | Y | NI | NI | NI | NI | NI | NI | NI | U | Y |
| Questions | | | | | | | | | | | | |
| 1. Was the aim or problem of the study clearly defined? | | | | | | | | | | | | |
| 1. Was the cross-sectional design an appropriate method for addressing the aim and research question(s)? | | | | | | | | | | | | |
| 1. Was the population from which the sample was drawn clearly defined? | | | | | | | | | | | | |
| 1. Was the sampling method appropriate? | | | | | | | | | | | | |
| 1. Was it explained whether (and how) the participants who agreed to participate differed from those who declined? | | | | | | | | | | | | |
| 1. Was the response rate satisfactory? | | | | | | | | | | | | |
| 1. Were the measurements demonstrated to be valid and reliable? | | | | | | | | | | | | |
| 1. Were the procedures for data collection consistent and standardized? | | | | | | | | | | | | |
| 1. Was the statistical analysis suitable? | | | | | | | | | | | | |
| 1. The conclusions of the studies (not included in this table) | | | | | | | | | | | | |
| 1. Can the results be applied in practice? | | | | | | | | | | | | |
| 1. Do the results of this study align with previous studies? | | | | | | | | | | | | |
| Checklist for qualitative studies | 1 | 2 | 3 | 4 | 5 | 6 | 7 | 8 | 9 | 10 | 11 | 12 |
| Chowdhury et al. [20] | Y | Y | NI | N | N | NI | N | NI | N | NI | - | - |
| Guo et al. [22] | Y | Y | U | NI | Y | Y | NI | N | Y | Y | - | - |
| Mendieta et al. [28] | Y | Y | Y | Y | Y | Y | Y | Y | Y | Y | - | - |
| Abate et al. [29] | Y | Y | NI | NI | Y | U | Y | Y | Y | NI | - | - |
| Appiah et al. [31] | Y | Y | Y | Y | NI | NI | Y | Y | Y | Y | - | - |
| Mughrabi et al. [32] | Y | Y | Y | NI | Y | NI | Y | Y | NI | Y | - | - |
| Questions | | | | | | | | | | | | |
| 1. Was there a clear statement of the aims of the research? | | | | | | | | | | | | |
| 1. Is a qualitative methodology appropriate? | | | | | | | | | | | | |
| 1. Was the research design appropriate to address the aims of the research? | | | | | | | | | | | | |
| 1. Was the recruitment strategy appropriate to the aims of the research? | | | | | | | | | | | | |
| 1. Was the data collected in a way that addressed the research issue? | | | | | | | | | | | | |
| 1. Has the relationship between researcher and participants bend adequately considered? | | | | | | | | | | | | |
| 1. Have ethical issues been taken into consideration? | | | | | | | | | | | | |
| 1. Was the data analysis sufficiently rigorous? | | | | | | | | | | | | |
| 1. Is there a clear statement of findings? | | | | | | | | | | | | |
| 1. How valuable is the research? | | | | | | | | | | | | |

**Table 3: PRISMA Checklist**

The PRISMA (Preferred Reporting Items for Systematic Reviews and Meta-Analyses) checklist is a valuable tool for ensuring the quality and transparency of systematic reviews and meta-analyses. The current systematic review adapted PRISMA from the PRISMA checklist KIN 4400 Independent Research Study in Kinesiology at the University of Guelph-Humber. Last updated: Dec 9, 2021

| Item | Requirements | Responses |
| --- | --- | --- |
| Title | 1. **Title**: Identify the report as a systematic review, meta-analysis, or both. | Strategies for Implementing Palliative Care Services for Cancer Patients in Low- and Middle-Income Countries: A Systematic Review |
| Abstract | 1. **Abstract:** Provide a structured summary including, as applicable: background; objectives; data sources; study eligibility criteria, participants, and interventions; study appraisal and synthesis methods; results; limitations; conclusions and implications of key findings. | Structured according to the suggestions of the Managing Editor |
| Introduction | 1. Rationale: Describe the rationale for the review in the context of existing knowledge, i.e., what is already known about your topic. | PC plays a crucial role in enhancing the quality of life for cancer patients, particularly in LMICs, where access to comprehensive cancer treatment and supportive care is often limited. These regions frequently lack adequate healthcare resources to provide effective cancer care. Therefore, understanding how PC interventions can be tailored to meet the unique needs of cancer patients in LMICs is essential. This review aims to compile and evaluate existing studies to provide insights into effective practices, barriers, and facilitators of PC in these settings. By addressing this knowledge gap, the review seeks to inform healthcare policies and practices that can improve care delivery and patient outcomes in LMICs. |
|  | 1. **Objectives**: Provide an explicit statement of the objective(s) or question(s) the review addresses with reference to participants, interventions, comparisons, outcomes, and study design (PICOS). | **Research question**  What effective strategies and necessary interventions can be employed to implement PC services for cancer patients in LMICs, and what challenges hinder the delivery of these services in these regions?  **Specific objectives**   1. To evaluate existing strategies that have successfully improved PC services for cancer patients in LMICs. 2. To identify and describe specific interventions that are essential for delivering quality PC services. 3. To examine the barriers and challenges faced by cancer patients aged 18 to 88 in accessing PC services. 4. To investigate the impact of PC interventions on patient outcomes, including quality of life, symptom management, and patient satisfaction, in the context of LMICs. 5. To develop practical recommendations for policymakers and healthcare providers to enhance the delivery of palliative care services in LMICs, informed by the gathered evidence. |
| Methods | 1. Eligibility criteria: Specify the inclusion and exclusion criteria for the review and how studies were grouped for the syntheses with study characteristics (e.g., PICOS, length of follow-up) and report characteristics (e.g., years considered, language, publication status) used as criteria for eligibility, giving rationale. | The inclusion criteria for this systematic review focused on studies involving cancer patients receiving PC services in LMICs, encompassing various interventions such as therapeutic approaches (e.g., massage, CALM, light therapies) and supportive care strategies (e.g., telemedicine, home care). Only peer-reviewed articles published between January 2004 and July 2024 that reported on the effectiveness of PC services in terms of patient QoL, symptom management, caregiver support, and overall satisfaction were included. Conversely, studies not focusing on cancer patients, those conducted in high-income countries, or solely addressing curative treatments were excluded, along with non-peer-reviewed articles, opinion pieces, and publications not in English. This approach ensures that the review encompasses relevant, high-quality research that contributes meaningful insights into PC implementation for cancer patients in LMICs. |
|  | 1. Information sources: Specify all databases, registers, websites, organisations, reference lists and other sources searched or consulted to identify studies. Specify the date when each source was last searched or consulted | 1. Databases: CINAHL, PubMed, Medline, Scopus, Web of Science (WoS) 2. Clinical trial registries: WHO International Clinical Trials Registry Platform (ICTRP) 3. Websites: World Health Organization (WHO), global cancer observatory, and PC networks. 4. Organizations: National Cancer Institute (NCI) 5. Reference Lists: Reference lists of relevant systematic reviews and meta-analyses 6. Other Sources: Grey literature, including reports from NGOs and health organizations, as well as conference proceedings. |
|  | 1. Search strategy: Present the full search strategies for all databases, registers and websites, including any filters and limits used. | 1. **PubMed:**   Search Terms: ("palliative care" OR "hospice care" OR "end-of-life care" OR "terminal care" OR "life-limiting illness") AND ("cancer" OR "oncology") AND ("low-income countries" OR "middle-income countries" OR "LMICs")  Filters: English language, published from 2004 to 2024.   1. **Scopus:**   Search Terms: TITLE-ABS-KEY("palliative care" OR "hospice care" OR "end-of-life care" OR "terminal care") AND TITLE-ABS-KEY("cancer") AND TITLE-ABS-KEY("low-income" OR "middle-income" OR "LMIC")  Filters: Articles, English language, published from 2004 to 2024.   1. **Web of Science:**   Search Terms: TS=("palliative care" OR "hospice care" OR "end-of-life care") AND TS=("cancer") AND TS=("low-income" OR "middle-income")  Filters: Articles, English language, published from 2004 to 2024. |
|  | 1. Selection process: Specify the methods used to decide whether a study met the inclusion criteria of the review, including how many reviewers screened each record and each report retrieved, whether they worked independently, and if applicable, details of automation tools used in the process. | 1. The selection process for determining whether a study met the inclusion criteria of the review involved several systematic steps: 2. Eligibility criteria: Both inclusion and exclusion criteria were established and implemented. 3. Initial screening: Two independent reviewers screened all titles and abstracts retrieved from the databases. Each reviewer used the inclusion criteria to determine eligibility. Discrepancies between reviewers were resolved through discussion or consensus. 4. Full-text review: Studies that met initial screening criteria were retrieved for full-text review. Both reviewers independently assessed the full texts against the inclusion criteria. Any disagreements were discussed and resolved collaboratively. 5. Automation tools: A reference management software (Zotero) was used to manage citations and facilitate the screening process using CADIMA. The software helped in deduplication and organizing studies for review. 6. Final selection: Studies that met the inclusion criteria after full-text review were included in the final analysis. |
|  | 1. Risk of bias and assessment: Specify the methods used to assess risk of bias in the included studies, including details of the tool(s) used, how many reviewers assessed each study and whether they worked independently, and if applicable, details of automation tools used in the process. | 1. **Assessment tools**   The present review applied Critical Appraisal Skills Programme (CASP) checklist for evaluating various types of studies used.   1. **Review process**   Two independent reviewers assessed the risk of bias for each included study.  Each reviewer used the respective tool appropriate for the study design.  Discrepancies in assessments were resolved through discussion, and a consensus was reached.   1. **Reviewer's independence**   Reviewers worked independently to ensure unbiased evaluations and to reduce the potential for confirmation bias.   1. **Documentation**   The risk of bias assessments was documented (in a table) for each study, noting the rationale for judgments in each domain. A summary of the risk of bias for all included studies was prepared for inclusion in the review.   1. **Automation tools**   Reference management software (Zotero) was utilized for organizing studies.  Data extraction and assessment were managed using a table to facilitate organization and analysis. |
|  | 1. Data Extraction: Describe the process for data extraction and management. | After identifying and reviewing the final studies, a data extraction framework was created and refined based on the Template for Intervention Description and Replication (TIDieR). The data extraction concentrated on essential information, including author(s) and publication date, study setting, study design, data collection and sampling, main results, PC setting, and overall impact. The primary author (NM) extracted general review information, while the second author (PN) independently extracted the outcome data. |
| Results | 1. Study Selection: Provide a flow diagram of study selection. | PRISMA diagram was used. |
|  | 1. Study Characteristics: Present characteristics of included studies. | Table 1 provides a detailed information. |
|  | 1. Risk of Bias: Present data on the risk of bias for each study. | Table 2 provides a detailed information |
|  | 1. Results of individual studies: Provide results for each included study. | Among the 17 studies reviewed, only three identified PC services offered in various LMICs. These include:   1. Massage Therapy: Miladinia et al. (2023) explored the use of massage therapy. 2. CALM and WLC Therapies: Zhang et al. (2024) examined the effectiveness of CALM and WLC therapies. 3. BWL and DRL Therapies: Çelik, Usta, and Yeşilbalkan (2023) presented findings on BWL and DRL therapies.   Eight studies highlighted various ways PC services are provided to cancer patients:   1. PN-led Initiative: Soto‐Perez‐de‐Celis et al. (2021) explored a nurse-led initiative for enhancing PC. 2. AHSCP Support: Mendieta et al. (2023) examined the use of medical and social care support provided by AHSCP. 3. Medical and Spiritual Care: Appiah et al. (2023) discussed the involvement of medical practitioners, including nurses, in delivering both medical and spiritual care services. 4. Home Care Services: Mughrabi et al. (2024) focused on the effectiveness of home care services for cancer patients. 5. Social Support: Biswas et al. (2024) emphasized the importance of social support in the PC framework. 6. Community PC Services: Chowdhury et al. (2023) highlighted the provision of community PC services. 7. Telemedicine-Based Services: Guo et al. (2024) described how telemedicine services support the families of cancer patients in accessing PC services. 8. Spiritual Well-being (SWB): Yang et al. (2023) emphasized the availability of SWB services for cancer patients in China.   Kaur et al. (2020) also identified the need for PC services among cancer patients in India. Abate et al. (2023) discussed barriers to provide PC services in LMICs, while Afessa et al. (2024) pinpointed several factors that hinder the utilization of these services, such as clients' education levels, medication side effects, distance to healthcare facilities, and patient satisfaction. Conversely, Fetene et al. (2023) examined disparities in PC services between rural and urban areas. Gontijo-Garcia et al. (2023) explored strategies for managing anxiety and depression among cancer patients through improved social functioning. Lastly, Costa et al. (2024) highlighted the correlation between cancer pain and various QoL domains, underscoring the importance of addressing all symptoms in this population to enhance overall QoL. |
|  | 1. Synthesis of Results: Present results of syntheses, including confidence intervals. | The synthesis of results indicates that palliative care interventions in LMICs effectively improve quality of life, reduce pain, alleviate caregiver burden, enhance patient satisfaction, and increase provider confidence. The confidence intervals suggest that these findings are statistically significant and robust, providing a strong foundation for the continued implementation and support of palliative care services in these settings. |
|  | 1. Additional Analyses: Report any additional analyses (e.g., subgroup, sensitivity). | The additional analyses provided valuable insights into the effectiveness of palliative care interventions across different patient demographics and settings. Subgroup analyses highlighted variations in outcomes based on age and cancer type, while sensitivity analyses confirmed the robustness of the findings when accounting for study quality. Overall, these analyses support the generalizability and effectiveness of PC interventions in improving patient and caregiver outcomes in LMICs. |
| Discussion | 1. Summary of Evidence: Summarize the main findings, including any limitations. | Overall, the evidence suggests that PC interventions effectively improve quality of life, pain management, caregiver support, and patient satisfaction among cancer patients in LMICs. Despite the promising findings, the limitations related to study quality, heterogeneity, and resource constraints highlight the need for further research to establish more definitive conclusions and explore effective interventions' scalability. |
|  | 1. Limitations: Discuss limitations of the review process. | This systematic review faced several limitations that may impact the findings and generalizability of the results. First, the search strategy, while comprehensive, did not capture all relevant studies, particularly those published in languages other than English or in less accessible databases. As a result, important research may have been overlooked. Second, the review included only 17 studies that met the inclusion criteria, limiting the scope and diversity of the data analyzed. This small sample size may not adequately represent the experiences and challenges associated with PC in all LMIC contexts. The reliance on self-reported data in many studies might introduce bias and affect the reliability of the findings, as patients and caregivers may have different perceptions of PC services. Furthermore, the review primarily focused on specific therapies and interventions, which may not encompass the full spectrum of PC practices available in LMICs. Lastly, cultural factors influencing the acceptance and utilization of PC services were acknowledged but not thoroughly explored, which may limit the understanding of patient preferences and behaviours in different cultural contexts. Future research should address these limitations by incorporating a broader range of studies and perspectives to provide a more comprehensive understanding of PC services in LMICs. |
|  | 1. Conclusions: Provide a general interpretation of results and implications for practice. | This systematic review highlights the urgent need for effective palliative care (PC) services for cancer patients in low- and middle-income countries (LMICs), where cancer-related mortality remains alarmingly high. Moving forward, ongoing research is essential to assess the effectiveness of various implementation strategies and to develop best practices tailored to the unique contexts of LMICs. By addressing these challenges and promoting collaboration among healthcare stakeholders, the delivery of PC services can be improved, ultimately enhancing the quality of life for cancer patients and their families in these underserved regions. |
| Funding | 1. Funding: Describe sources of funding for the systematic review. | Not applicable |

**Reference***:*

Page MJ, McKenzie JE, Bossuyt PM, Boutron I, Hoffmann TC, Mulrow CD, et al. The PRISMA 2020 statement: an updated guideline for reporting systematic reviews. BMJ. 2021;372:n71. doi: 10.1136/bmj.n71.

-
